# Weakly supervised lesion localization for age-related macular degeneration detection using optical coherence tomography images

**DOI:** 10.1371/journal.pone.0215076

**Published:** 2019-04-05

**Authors:** Hyun-Lim Yang, Jong Jin Kim, Jong Ho Kim, Yong Koo Kang, Dong Ho Park, Han Sang Park, Hong Kyun Kim, Min-Soo Kim

**Affiliations:** 1 Department of Information and Communication Engineering, Daegu Gyeongbuk Institute of Science & Technology (DGIST), Daegu, Republic of Korea; 2 Department of Ophthalmology, School of Medicine, Kyungpook National University, Daegu, Republic of Korea; University of Florida, UNITED STATES

## Abstract

Age-related macular degeneration (AMD) is the main cause of irreversible blindness among the elderly and require early diagnosis to prevent vision loss, and careful treatment is essential. Optical coherence tomography (OCT), the most commonly used imaging method in the retinal area for the diagnosis of AMD, is usually interpreted by a clinician, and OCT can help diagnose disease on the basis of the relevant diagnostic criteria, but these judgments can be somewhat subjective. We propose an algorithm for the detection of AMD based on a weakly supervised convolutional neural network (CNN) model to support computer-aided diagnosis (CAD) system. Our main contributions are the following three things. (1) We propose a concise CNN model for OCT images, which outperforms the existing large CNN models using VGG16 and GoogLeNet architectures. (2) We propose an algorithm called Expressive Gradients (EG) that extends the existing Integrated Gradients (IG) algorithm so as to exploit not only the input-level attribution map, but also the high-level attribution maps. Due to enriched gradients, EG can highlight suspicious regions for diagnosis of AMD better than the guided-backpropagation method and IG. (3) Our method provides two visualization options: overlay and top-*k* bounding boxes, which would be useful for CAD. Through experimental evaluation using 10,100 clinical OCT images from AMD patients, we demonstrate that our EG algorithm outperforms the IG algorithm in terms of localization accuracy and also outperforms the existing object detection methods in terms of class accuracy.

## Introduction

Deep learning is of growing importance in many applications, such as image recognition and image localization. A number of efforts have been made to classify medical images with regard to disease using deep learning models, and accordingly, the explainability of such models has become an important topic of research in the medical imaging field. Because of the critical nature of medical applications, not all decisions should be left to models. As in previous research such as [1–3], it is safer to use the models only to support clinicians’ decisions.

Age-related macular degeneration (AMD) is the leading cause of irreversible blindness in people 50 years of age or older in the developed world. It is known that damage to the retinal pigment epithelium and a chronic inflammatory response can lead to deposit yellow or white accumulations of extracellular material between Bruch’s membrane and RPE and develop choroical neovascularization (CNV) or retinal atrophy. The expression of angiogenic cytokines such as vascular endothelial growth factor can also induce retinal degeneration [4]. Most of these changes can be detected by taking macular images using optical coherence tomography (OCT). OCT is also a critical modality for retinal evaluation before the initiation of anti-VEGF therapy and for the assessment of the subsequent therapeutic effect [5]. Interpretation of OCT images is usually performed by a clinician, and it can aid in the diagnosis and selection of treatment modalities of AMD on the basis of the relevant criteria. However, these judgments can require a great deal of human efforts and be somewhat subjective. Thus, an accurate computer-aided diagnosis (CAD) system for AMD detection is needed for resolving this situation.

There have been proposed a number of methods to diagnose AMD using OCT images [6–9]. In particular, the deep learning-based methods, [6] and [7], have utilized well-known convolutional neural network (CNN) models, VGG16 [10] and GoogLeNet [11], and achieved the accuracies of 93.45% and 94%, respectively. However, they only can do prediction, but cannot localize suspected AMD lesions in OCT images and so might not be very useful as a CAD system. In addition, they can predict only two or three classes, but clinicians in hospitals require predicting four classes in many cases, normal, dry AMD, wet AMD (observation only) and wet AMD (anti-VEGF injection required), which are more difficult to discriminate among.

Meanwhile, several algorithms [12–15] have been proposed to explain what a model considers and predicts from input images. In particular, the guided-backpropagation method [14] and the Integrated Gradient (IG) algorithm [15] have been proposed to find the attributes of the input that most strongly contribute to predicting the class of the input data. For image data, they can find an *attribution map*, in which the pixels in the image that are important for prediction are highlighted. For the attribution map, guided-backpropagation calculates the pixels that have a positive effect on a class label by using the gradients of the model and considering the activation functions of the model. On the other hand, IG integrates all the gradients computed at the points along the path from the input image vector to the baseline (e.g., black) image vector. In general, IG calculates and exploits more gradients than guided-backpropagation to find the attribution map. These methods have been proven to be effective for general images like ImageNet. However, they tend to be less effective for medical images of relatively small amount of information, e.g., OCT images.

In this paper, we propose an end-to-end weakly supervised deep learning-based method for predicting the class of AMD and locating its lesions in OCT images. The term *weakly supervised* means that our algorithm only uses weakly-labeled (i.e. image-level labeled) datasets which do not contain any region information to localize lesions in images. That is, our method does not need any bounding box information, unlike object detection methods [16, 17]. *End-to-end* means that our method can be performed and improved jointly according to the performance of the CNN model used.

The proposed method consists of the following two components: a new concise CNN model for OCT images and so-called the Expressive Gradients (*EG*) algorithm. Our CNN model outperforms the existing models for AMD detection regardless of its 20X fewer parameters. Our EG algorithm exploits not only the gradients with respect to the input image, but also the gradients with respect to all the intermediate feature maps, for conjugating gradient backpropagation as much as possible. From such enriched gradients, we can find good attribution maps in the images having relatively small amount of information (e.g., OCT images). As a result, it can localize the lesions better than the conventional guided-backpropagation method and the IG algorithm, which are exploiting only the gradients with respect to input image, for OCT images. It improves both coverage and hit rate compared with the guided-backpropagation method and IG algorithm. To support the field compatible CAD system, our method provides two kinds of visualization options: image overlay and bounding boxes. For the latter option, the number of boxes (i.e., top-*k*) can be controlled by clinicians.

## CNN model for OCT images

In this section, we present our CNN model for predicting the presence of AMD from OCT images. Since our EG algorithm is solely based on the weights and gradients of the CNN model used for explaining the lesions, it is important to use a concise CNN model of a higher accuracy for better explainability. However, most of the existing CNN models for OCT images are built using the CNN architectures for general image datasets like ImageNet, and so, tend to be very large and contain unnecessary weights and features for explaining the lesions of AMD. Thus, we propose a concise and accurate CNN model for OCT images.

**Fig 1:**
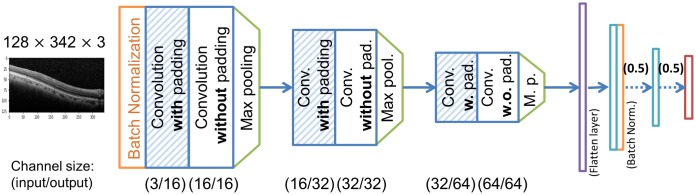
The architecture of our CNN model for AMD prediction on OCT images.

Fig 1 presents the architecture of our CNN model, which consists of six convolutional layers and four dense layers. The last layer contains four neurons, which correspond to the four classes for input OCT images. We acquire a total of 10,100 clinical OCT images from a national university hospital for training and testing our model and other models, where are 5,075 normal images, 2,225 dry AMD images, 650 wet AMD (observation only) images, and 2,150 wet AMD (anti-VEGF injection required) images. For the images, each macular scan was performed as vertical and horizontal 25-line raster macular scan from 224 patients over age of 50, and every image was extracted from each macular OCT scan. The dimension of an original image is about of 380 × 1000 of RGB (i.e., three) channels, which does not contain personal information such as name. Our model takes a downscaled image having the dimension of 128 × 342 of RGB channels. We use the same three channels for the input of the model although the original input images look like gray-scale ones since we consider using the model for the CAD system. We perform batch normalization to achieve the robustness for the case in which a batch is biased toward a specific class and to prevent gradient vanishing. Our model is composed of three convolutional blocks, each of which has two consecutive convolution layers followed by a max pooling layer. The former convolution layer is with padding, while the latter one is without padding. The kernel sizes are of 3 × 3, and the stride is one. The first dense layer is the flattening layer, and the numbers of neurons in the last three dense layers are 200, 20, and 4, sequentially. Right after the second dense layers, there is a batch normalization layer. In the last two connections, dropout operations with 0.5 are applied. We use the ReLU as activation function for all the layers except the output layer, where we use the sigmoid. The channel size of each convolutional layer are listed in Fig 1.

For evaluation, we separate a total of 525 images of eight patients from 10,100 input images. We randomly selected those eight patients for separate test data, in particular, two normal patients, two dry AMD patients, one wet AMD (observation only) patient, and three wet AMD (anti-VEGF injection required) patients. We use 10, 100 − 525 = 9,575 images for training both our model and other existing models. For training data, we track the validation loss and accuracy using 5-fold cross-validation.

For training our model, we use a cross-entropy loss function and the Adam optimization algorithm. We initialize the weights using the Xavier initialization [18]. We note that the model is trained using only OCT images with their class labels, without additional information or supervision such as bounding boxes or segmentation information. We perform a total of 200 epochs, where we use the learning rates of 0.01, 0.001, and 0.0001, for the first 100 epochs, the next 50 epochs, and the last 50 epochs, respectively. The batch size for both training and validation is 101.

Table 1 shows the accuracies of the existing models for predicting AMD severity labels for OCT images and our proposed model. Since most of the existing models are evaluated using their own data, which are not publicly available, we evaluate those models using our data. In detail, we consider the models in [6–9], where [8, 9] are non-deep learning models, and [6, 7] are deep learning ones. Among the models, we cannot evaluate the model in [9] since there is no codes available and no information for implementation such as the dictionary of visual words which they used for preprocessing of OCT images. Deng et al. [8] uses a histogram of 11 bins from 40 Gabor filters and feeds the abstracted features of images to the classifier. Lee et al. [6] uses the VGG16 architecture of 130 M parameters with the Xavier initialization. Karri et al. [7] uses the GoogLeNet model of 6.8 M parameters with transfer learning. For all the models in [6–8], we modified their output layer such that they can predict four classes instead of two or three classes since our data is of four classes. We train those models using the same 9,575 training images with tracking the validation accuracy using 5-fold cross-validation and test using the same 525 separate test images.

**Table 1:** Performance evaluation of AMD prediction models for OCT images (STD = standard deviation).

Method	Performance on their data			Performance on our data		
	# of classes to predict	# of images	Reported accuracy	Test accuracy	Validation accuracy	5-fold CV STD
VGG16 [6]	2 (Normal, AMD)	2.6M	93.45%	71.81%	80.70%	0.0154
RF^‡^ with BoW^§^ [9]	5 (Normal, Early AMD, Intermediate AMD, Advanced AMD GA, Advanced AMD CNV	3,265	80.4%	-	-	-
RF with GFET^¶^ [8]	3 (Normal, Dry AMD, Wet AMD)	420	88.7%	49.25%	57.92%	0.0089
SVM with GFET^¶^ [8]	3 (Normal, Dry AMD, Wet AMD)	420	94.4%	51.50%	62.28%	0.0147
NN with GFET^¶^ [8]	3 (Normal, Dry AMD, Wet AMD)	420	78.1%	52.50%	51.81%	0.0902
GoogLeNet [7]	3 (Normal, Dry AMD, DME^#^)	3,231	94%	80.18%	82.61%	0.0182
**Our model**	4 (Normal, Dry AMD, Wet AMD with observation only, Wet AMD with anti-VEGF injection required)	9,575 (training), 525 (testing)	-	**94.86%**	**96.05%**	0.0035

^‡^RF: Random Forest,

^§^BoW: Bag of visual Words,

^¶^GFET: Gabor Filtering Energy Transform,

^#^DME: Diabetic Macular Edema.

Table 1 shows the performance of the existing models on both their data and our data and the performance of our model. Our model achieves 94.86% test accuracy and 96.05% validation accuracy with the standard deviation 0.0035. The full list of validation accuracies is 95.55%, 96.48%, 95.87%, 96.20% and 96.15%. It outperforms the existing models, in particular, 49.25-52.50% in [8], 71.81% in [6], and 80.18% in [7] in terms of test accuracy. The reported accuracies of the existing models in Table 1 all are the validation accuracies on their own data. Moreover, these accuracies are the results when the number of output classes is only two or three. On the contrary, our data is much more complex than the data used in the existing studies since it consists of four classes which are more difficult to discriminate among. These results suggest that constructing a new concise CNN model for complex OCT images can be more effective for AMD detection than using the well-known models constructed for general images such as VGG16 or GoogLeNet. OCT images usually look gray, that is, the amount and variety of information in the images are much smaller than those of general images such as ImageNet. In addition, the class patterns (i.e., lesions) in OCT images are usually subtle to discriminate compared with those in general images.

## Expressive Gradients (*EG*) algorithm

EG is a fully weakly supervised localization algorithm for finding suspected AMD lesions in OCT images. The conventional guided-backpropagation method [14] and the IG algorithm [15] exploit the backpropagation of gradients, in particular, the gradients with respect to the input image. However, this approach tends to lose a considerable amount of gradient information during backpropagation as a neural network model has more ReLU and maxpooling layers or becomes deeper. ReLU solves the gradient vanishing problem of the sigmoid activation function, but has a dying ReLU problem [19] where a neuron is not longer learned once its value becomes zero. The zero values of dead neurons are propagated to the next layers, and so, their gradients are not available during backpropagation, which can degrade the explainability of guided-backpropagation and IG that exploit only the gradients with respect to the input image. The low quality of OCT images, that is, a relatively small amount and variety of information, worsens this tendency. To alleviate this problem, our EG algorithm exploits not only the gradients with respect to the input image, but also the gradients with respect to all the intermediate feature maps. This proposed approach can be very useful for conjugating the gradient backpropagation as much as possible even for the medical images of low quality.

**Fig 2:**
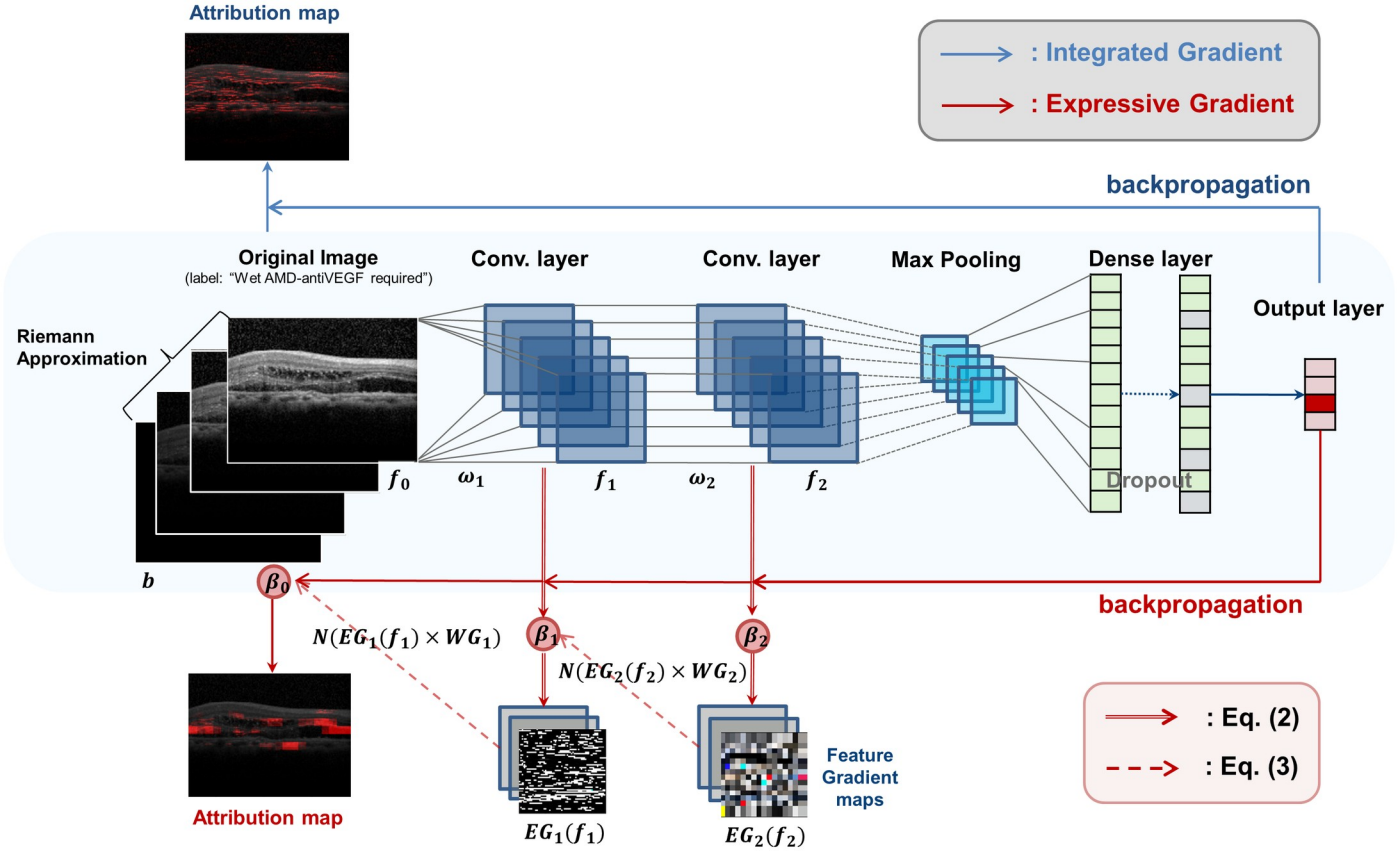
Operations of Integrated Gradients and Expressive Gradients.

The conventional IG algorithm calculates the attribution map as in Eq (1) [15], where *x* is an input image, *b* the baseline image, and *F*: *R*^*n*^ → [0, 1, 2, 3] the CNN model classifying *x* into four classes. Here, the baseline image is the input image satisfying *F*(⋅) = 0. The IG algorithm calculates an attribution map by integrating all computed gradients at all points along the path from the input image vector to the baseline image vector. In Fig 2, the blue line above the CNN model indicates the operation flow of the IG algorithm.

$$\begin{array}{c} IG\left(x\right)=\left(x-b\right)\times \int_{\alpha =0}^{1}\frac{\partial F(b+\alpha \times (x-b))}{\partial x}d\alpha \end{array}$$ (1)

The EG algorithm can be formulated as in Eqs (2) and (3), where Eq (2) means calculation of an attribution map for a given feature map (*f*_*i*_), and Eq (3) means calculation of the expressive gradients. In Eq (2), *b* indicates the baseline image, *f*_*i*_ the feature map from the *i*-th convolution layer, and *F*_*i*_ the partial CNN model consisting of all the layers from the *i*-th convolution layer to the last layer of the model. We note that Eq (2) does not consider pooling layers, but only considers convolution layers, as the IG algorithm does. For instance, in Fig 1, *f*_3_ is the feature map of the third convolution layer of the channel size (16/32), and *F*_3_ indicates the partial CNN model from the third convolution layer to the output layer. Here, *f*_0_ means the input image, and *F*_0_ the entire CNN model.

$$\begin{array}{c} EG_{i}\left(f_{i}\right)=\left(f_{i}-b\right)\times \int_{\alpha =0}^{1}\frac{\partial F_{i+1}\left(b+\alpha \times \left(f_{i}-b\right)\right)}{\partial f_{i}}d\alpha \end{array}$$ (2)

Intuitively, EG in Eq (3) is calculated as a weighted sum of the attribution maps {*EG*_*i*_(⋅)} of all feature maps {*f*_*i*_}. In Eq (3), *L* indicates the number of convolution layers in the model, *N*(⋅) the 0-to-1 normalization of a given attribution map, and the *β*_*i*_ the hyperparameters that determine the weights of each normalized attribution map (0 ≤ *β*_*i*_ ≤ 1). For the CNN model in Fig 1, *L* becomes six. *N*(⋅) makes the values in each attribution map in the same range. In Fig 2, the red solid line under the model indicates the operation flow of EG algorithm, the red double line Eq (2), and the red dotted line Eq (3).

Eq (3) performs the summation of the attribution maps having different channel dimensions. Since each feature map *f*_*i*_ has a different channel dimension, and *EG*_*i*_(*f*_*i*_) has the same dimension with *f*_*i*_, {*EG*_*i*_(⋅)} cannot be summed directly. Thus, we reduce the number of channels of all the intermediate attribution maps to the same dimension, in particular, three (i.e., RGB) for the visualization of those maps on the input images. We introduce the term *WG*_*i*_([: *ω*_*i*_]) (*i* > 0) in Eq (3) in order to change the dimension of each attribution map. Let *ω*_*i*_ be the weights between the (*i* − 1)-th and *i*-th layers, *d*_*i*−1_ be the dimension of (*i* − 1)-th layer, and *d*_*i*_ be the channel dimension of *i*-th layer. Then, $\omega_{i}\in R^{d_{i},d_{i-1},m,m}$, where *d*_*i*_ indicates the output dimension of *ω*_*i*_, *d*_*i*−1_ the input dimension of *ω*_*i*_, and *m* × *m* the kernel size. We define *W*_*i*_ as a series of multiplications of *ω*_*i*_ from the input layer to the *i*-th layer with marginalization of the kernel, i.e., $W_{i}=\prod_{l=0}^{i}\sum^{m,m}\omega_{l}^{d_{l},d_{l-1},m,m}$. We note that $W_{i}\in R^{d_{i},3}$, since *d*_0_ = 3 (i.e., RGB). Then, we define *WG*_*i*_ as a transpose of *W*_*i*_, i.e., $WG_{i}=W_{i}^{T}$, which can be used to convert the dimensions of the attribution maps to the dimension of input image.

$$\begin{array}{c} EG=\sum_{i=0}^{L}\beta_{i}\times N\left(EG_{i}\left(f_{i}\right)\times WG_{i}\left(\left[:\omega_{i}\right]\right)\right) \end{array}$$ (3)

Algorithm 1 presents the pseudo code for computing *N*(*EG*_*i*_(*f*_*i*_) × *WG*_*i*_([: *ω*_*i*_])) in Eq (3), which also considers not only convolution layers, but also pooling layers. It first calculates the attribution map *attMap* for a feature map *f*_*i*_ as in Eq (2) (Line 1). Then, it adjusts the dimension of *attMap* by multiplying *attMap* with the marginalized weights of *ω*_*i*_, i.e., *wg* (Lines 5-7), or upools *attMap* for dealing with a pooling layer (Lines 3-4), until it reaches the input layer (Line 2). The unpooling operation in Line 4 is different from the conventional unpooling operation used in CNN backpropagation. To deal with maxpooling, we unpool the attribution map *without* a maxpooling index and copy the same value to all indexes. This allows us to preserve the information of higher-level attribution maps in all receptive fields in the input image. When calculating *EG*_*i*_ in Line 1, we approximate it through discretization as in the IG algorithm [15], in particular, by using 50 steps for the Riemann sum approximation.

**Algorithm 1**: Computing *N*(*EG*_*i*_(*f*_*i*_) × *WG*_*i*_([: *ω*_*i*_]))

 **Input**: *f_i_*, feature map of the *i*-th layer

   [: *ω_i_*], all weight matrices from *ω*_1_ to *ω_i_*

1 *attMap* ← *EG*_*i*_(*f*_*i*_);

2 **while** *i* > 0 **do**

3  **if** *d*_*i*_ == *d*_*i*−1_ **then**

4   Unpool *attMap*;

5  **else**

6   $wg\leftarrow \sum^{m,m}\omega_{i}^{d_{i},d_{i-1},m,m}$;

7   *attMap* ← *attMap* × *wg*;

8  **end**

9  *i* ← *i* − 1;

10 **end**

11 **return** *N*(*attMap*);

Based on the resulting attribution maps, we localize the lesions by the following three steps: finding the pixels in the attribution maps that have positive values; normalizing the attribution maps to the range [0, 1]; and finding the pixels larger than a given threshold *τ*. There are two options for visualization of the pixels found. The first option visualizes the highlighted image with an overlay. The second option visualizes the lesions as the top-*k* bounding boxes. In detail, we construct circles around the pixels found and draw the boxes containing the circles. We calculate a sum of pixel values for each bounding box and sort the boxes such that only the top-*k* boxes can be visualized by clinicians with interactively changing *k*. For the purpose of CAD, the first option, i.e., the overlay option would be preferred. We mainly use the bounding box option for performance comparison in this paper.

## Experimental evaluation

We set *b* in Eq (2) to black image and set the hyperparameters {*β*_*i*_} (0 ≤ *i* ≤ 6) to the values of [1, 0.166, 0.166, 0.166, 0.166, 0.166, 1]. If we give a high weight to the input attribution map, i.e., *β*_0_, the overlay visualization option tends to highlight broader areas of low intensity, and so, it becomes difficult to find clear and distinct bounding boxes. By contrast, giving a high weight to the high-level attribution map, e.g., *β*_6_, results in the overlay images that highlight biased intense areas and large bounding boxes. We give high weights to both *β*_0_ and *β*_6_ since it shows overall good results. We leave the optimization of hyperparameters for future work.

**Fig 3:**
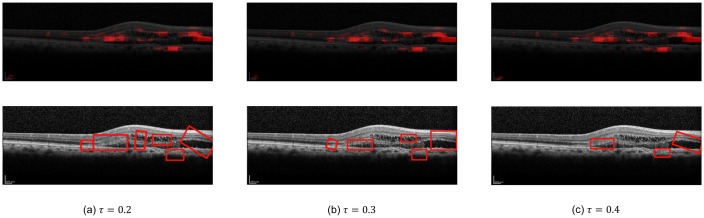
Variation of *τ* in wet AMD case.

For evaluation of our EG algorithm and other two methods (i.e., guided-backpropagation and IG), we use the same CNN model proposed in Fig 1 and trained using our 10,100 OCT images. Although the CNN model is trained using only class labels without annotations like bounding boxes, all three methods, guided-backpropagation, IG, and EG can localize the lesions as highlighted images or bounding boxes based on their resulting attribution maps. To measure the localization accuracy of those methods, we use a total of 1,057 images which two skilled ophthalmologists annotated dry AMD and wet AMD with bounding boxes based on disease judgments. The number of boxes contained in 1,057 images is 3,761. They are used for the ground truth to measure the localization accuracy. The boxes contain drusen, choroidal neovascularization membrane, subretinal fluid, intraretinal fluid, and intraretinal hyperreflective material. We use bounding-box-level annotation instead of pixel-level annotation since the latter is too difficult to obtain in high resolution OCT images. Moreover, we could obtain only 1,057 annotated images since it costs a lot for skilled ophthalmologists to annotate the images. All procedures have been supervised by an experienced retinal specialist. For measuring the localization accuracy, we define and use the *coverage* and the *hit rate*, which will be described in detail later. Here, we empirically set the threshold *τ*, the hyperparameter for determining the degree of highlighting, to 0.3 for our experiments. Fig 3 shows the results in the overlay and bounding box options while varying *τ* from our EG algorithm.

We conduct another kind of experiment, comparing our method with the existing object detection methods. In particular, we compare the class accuracy of our method with that of the well-known object detection methods, Single Shot Multibox Detector (SSD) [17] and Faster R-CNN [16]. We use the above 1,057 annotated images for training of the methods with 5-fold cross-validation. Here, we train our CNN model in Fig 1 using the 1,057 images with the class labels of the images. On the contrary, we train the detection methods using 3,761 bounding boxes in 1,057 images with the class labels of the bounding boxes. If we train the object detection methods and test their localization accuracy using the ground-truth bounding boxes through cross-validation, their accuracies would be very high (almost 100%) due to overfitting. In contrast, our method finds the bounding boxes for lesions based on the model trained not using any bounding box information at all. Thus, it would be unfair to compare our method with the object detection methods in terms of the localization accuracy. Instead, we can compare them in terms of the class accuracy since they all utilize class information when training their models.

### Localization accuracy

We compare our EG algorithm with the guided-backpropagation method and the IG algorithm by using the same CNN model in Fig 1, in terms of the localization accuracy. We evaluate the performance of our method both quantitatively and qualitatively. For quantitative evaluation, we use two measures, coverage and hit rate. They are formulated as in Eqs (4) and (5), where *N*_*pixel*_(*area*) is the number of pixels in a given area, *B*_*proposed*_ the set of the proposed bounding boxes, *B*_*ground*_ the set of the ground-truth bounding boxes, and the operator ∩ finds the intersection area between given two operands. In general, there are multiple bounding boxes indicating the lesions in each image.

$$\begin{array}{c} coverage=\frac{N_{pixel}\left(B_{proposed}\cap B_{ground}\right)}{N_{pixel}\left(B_{ground}\right)} \end{array}$$ (4)

$$\begin{array}{c} hit\;rate=\frac{N_{pixel}\left(B_{proposed}\cap B_{ground}\right)}{N_{pixel}\left(B_{proposed}\right)} \end{array}$$ (5)

Table 2 shows the results of the coverage and hit rate of the three methods. Our EG algorithm outperforms both guided-backpropagation and IG in terms of both coverage and hit rate. We note that the coverage and hit rate of our method are not so high due to the difference in between the way of making the ground-truth bounding boxes by ophthalmologists and the way of proposing the bounding boxes by our method. The ophthalmologists tend to make boxes largely such that even background pixels are contained in the ground-truth bounding boxes, whereas our method tends to propose the boxes compactly. In S4a and S4b Fig show that the ground-truth boxes contain lots of background pixels. The performance of the guided-backpropagation method in Table 2 is very poor, which seems to be due to the characteristics of the OCT images. Guided-backpropagation quickly extracts the positive gradient values by considering the relu activation function during forward pass and backward pass. Although the amount of computation of this approach is much smaller than those of IG and EG, it may be effective for general images, which contain a relatively large amount and variety of information. However, OCT images have a relatively small amount and variety of information, where the methods that can accumulate gradients like IG or amplify gradients like our EG can be more effective.

**Table 2:** Quantitative localization analysis (STD = standard deviation).

Method	Mean of *Coverage*	STD of *Coverage*	Mean of *Hit Rate*	STD of *Hit Rate*
Guided-backpropagation	0.076262	0.133901	0.071629	0.129572
Integrated Gradients (IG)	0.423445	0.307058	0.283803	0.240317
**Our method (** ***EG***)	**0.497719**	0.375928	**0.367342**	0.293104

**Fig 4:**
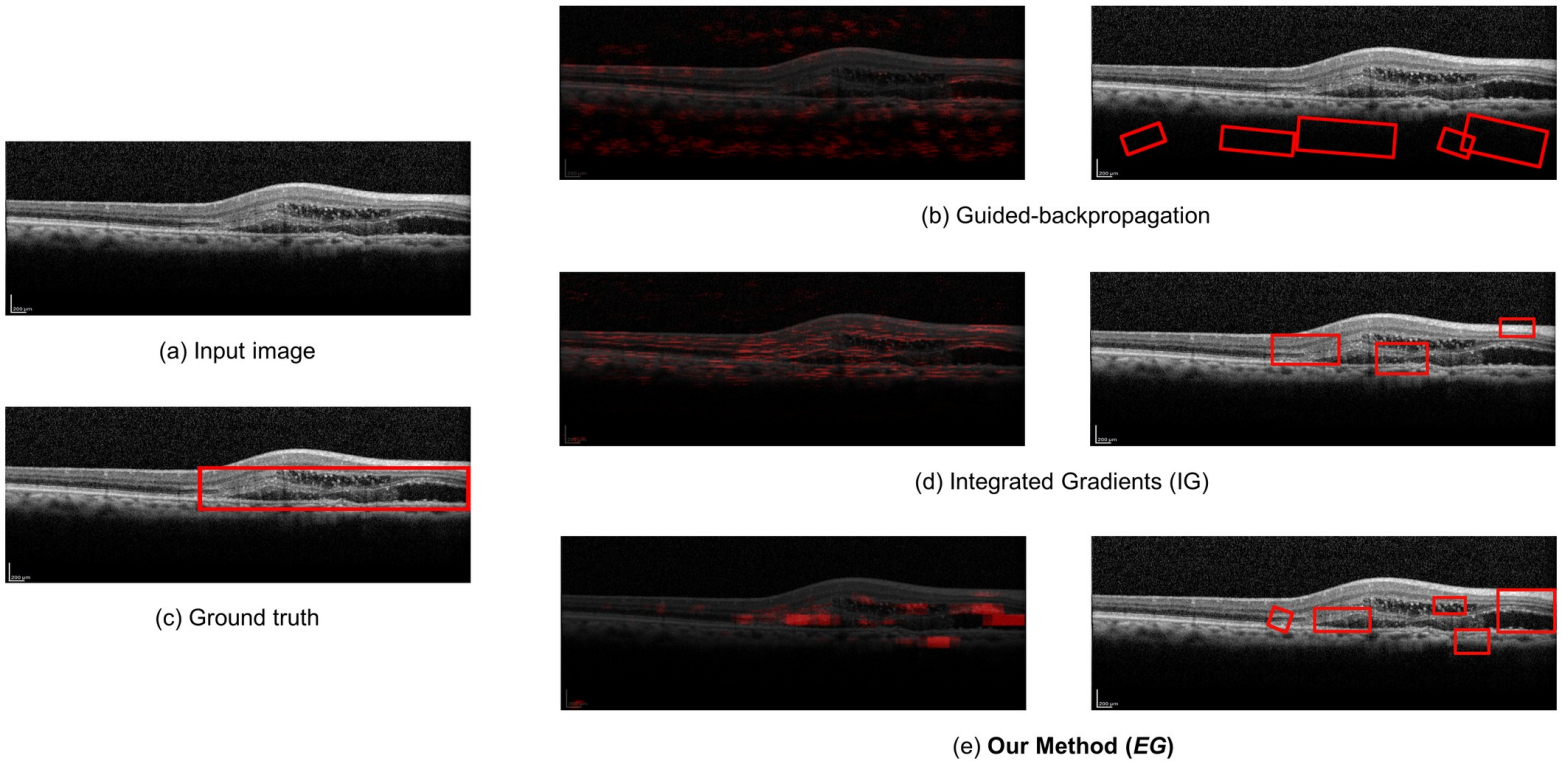
Qualitative analysis for the wet AMD (with anti-VEGF injection required) case.

**Fig 5:**
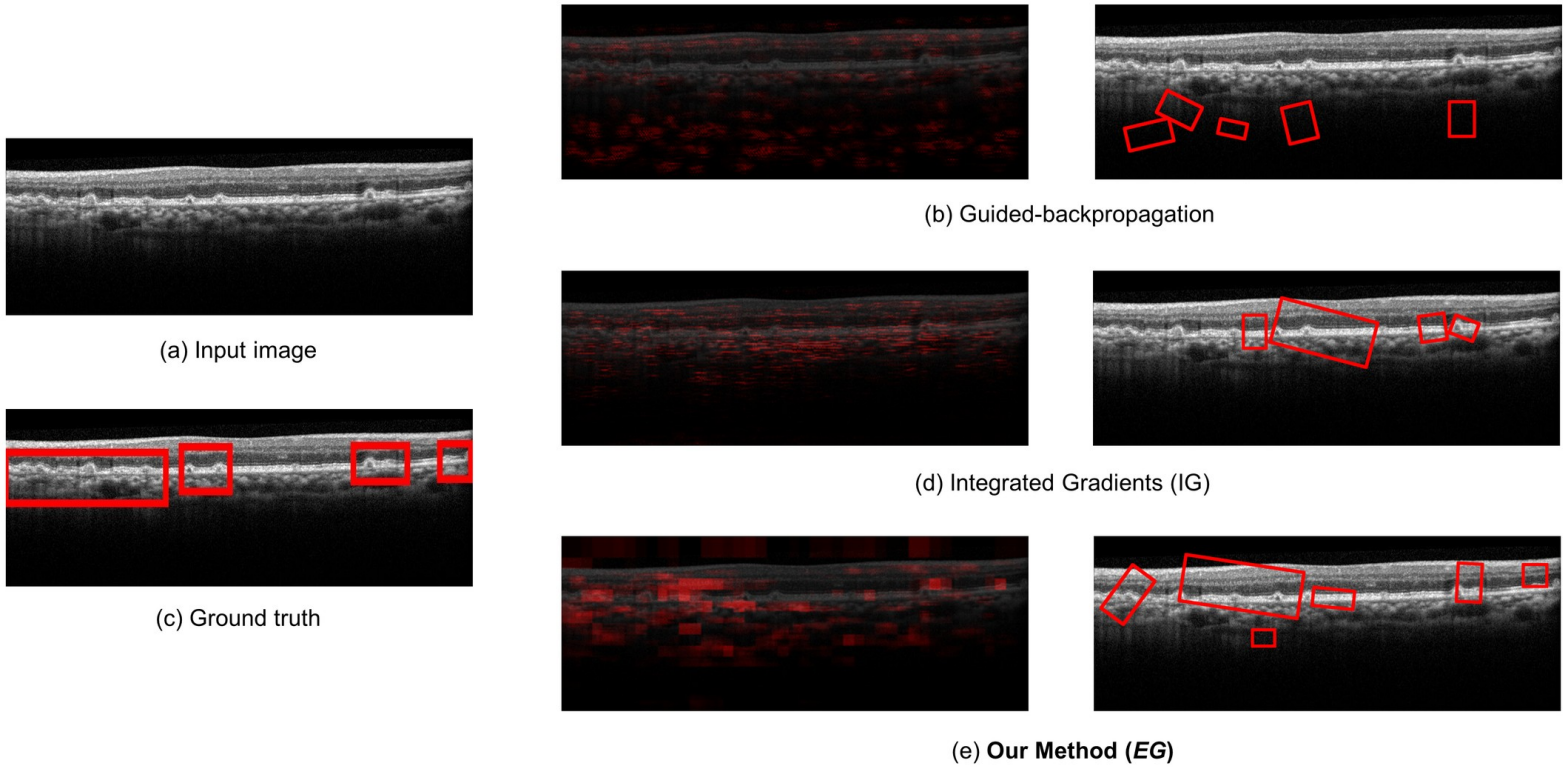
Qualitative analysis for the Dry AMD case.

For qualitative evaluation, we compare the overlay images and the bounding boxes found by guided-backpropagation, IG, and EG for various classes and images. Fig 4 shows the result for the wet AMD case with anti-VEGF injection, where our method produces a clearer and more specific overlay image than the IG algorithm, and at the same time, detects fluid in the wet AMD case where the IG algorithm cannot detect well. Guided-backpropagation usually localizes the background and cannot find the legion properly. Fig 5 shows the result for the dry AMD case, where our method can detect drusen in the image that the others cannot. In particular, the left side of the image is more highlighted than the other side. Such a difference in detection performance is that our EG algorithm exploits not only the input-level attribution map, but also the high-level attribution maps for detection and visualization of lesions. Since the prediction is done in the dense layers of the model based on the high-level features of a image, there is inherent benefit in using the high-level attribution maps, especially for medical images having a small amount of information. The more results for qualitative evaluation are presented in S1–S3 Figs.

### Class-level accuracy

**Fig 6:**
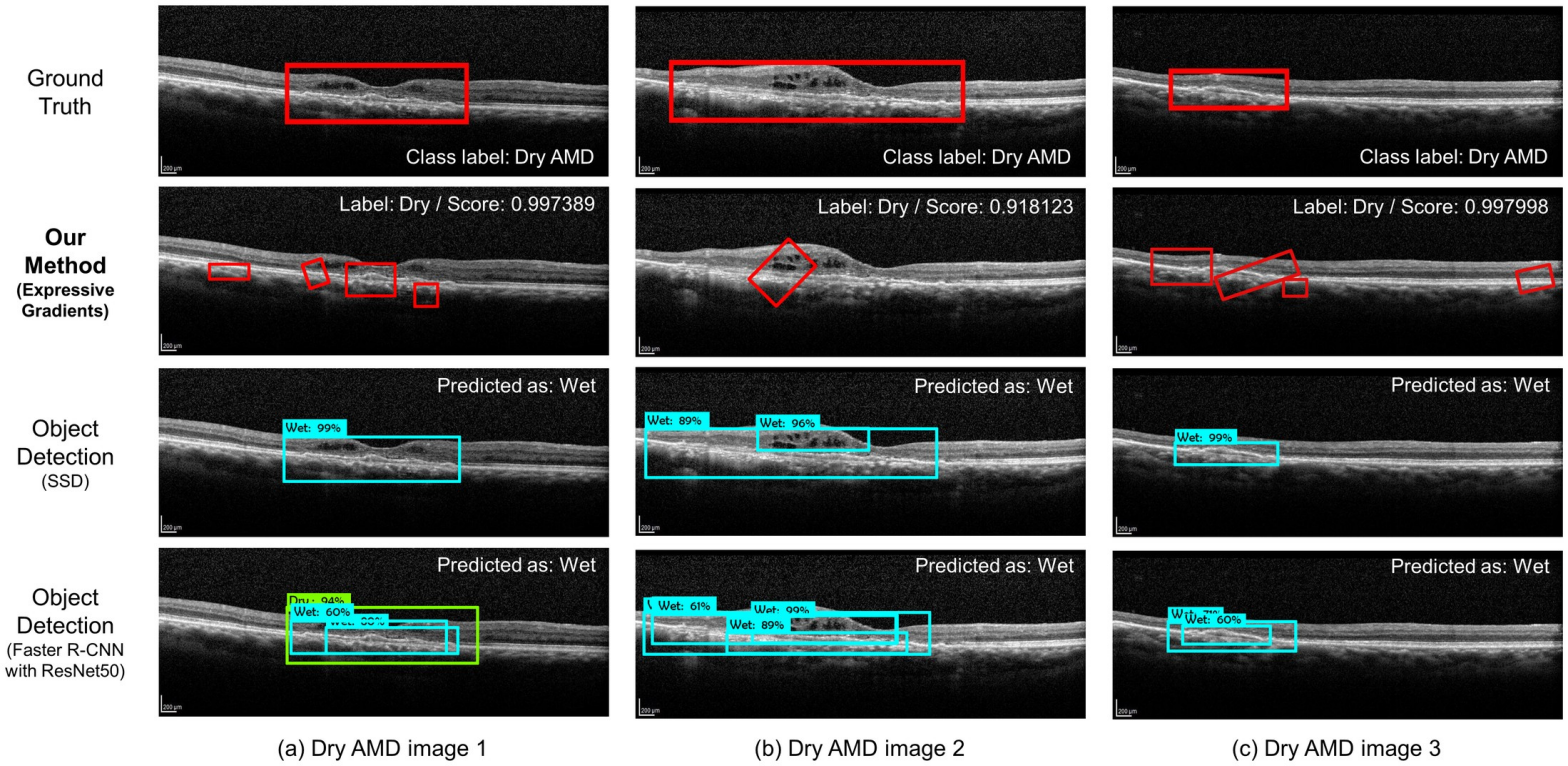
Misclassified images of the object detection methods.

We compare our method with the object detection methods, SSD [17] and Faster R-CNN [16], in terms of the class accuracy. We use the MobileNet [20] and ResNet50 models [21], which are widely used for the object detection methods, for SSD and Faster R-CNN, respectively, and train the models using 3,761 bounding boxes with their class labels. We use the Tensorflow object detection API [22] for implementing our experimental framework. SSD and Faster R-CNN may detect multiple objects with their class labels, and so, we decide the class label of a image by majority, i.e., the class label of the largest number of boxes. If two different class labels have the same number of boxes, we decide the class label of the box having the largest area. Table 3 shows the mean and standard deviation of validation accuracy with 5-fold cross-validation. The number of failures with no class in the table means the number of images that the method cannot detect any bounding box and so fail to decide the class label. In the results, our method significantly outperforms both SSD and Faster R-CNN in terms of class accuracy. For our data, we found that SSD and Faster R-CNN tend to predict the dry cases as wet cases incorrectly. Fig 6 shows the three images that the object detection methods fail to classify the label correctly. From the results, we can see that the object detection methods can detect the bounding boxes themselves more correctly than our method, but fail to identify what each box detected means in many cases.

**Table 3:** Comparison of class-level accuracy with object detection methods (STD = standard deviation).

Method	Class-level accuracy	5-fold CV STD	# of failures with no class
SSD	33.98%	0.1351	23 images
Faster R-CNN with ResNet50	68.96%	0.0923	9 images
**Our CNN model**	**94.55%**	0.0104	-

## Conclusions

In this paper, we have proposed a weakly supervised deep learning-based method for predicting the class of AMD and locating its lesions in OCT images. Our proposed CNN model for OCT images achieves a higher accuracy for AMD detection than the existing large CNN models. The compactness of our model is beneficial to the gradient-based methods such as EG algorithms since it can reduce the loss of gradients during backpropagation. Our EG algorithm outperforms the conventional guided-backpropagation method and IG algorithm in terms of coverage and hit rate due to its exploitation of high-level attribution maps. Our method also can localize lesions only using class labels without ground-truth bounding boxes. To the best of our knowledge, our method is the first method to localize AMD lesions for OCT images in a weakly supervised manner. It has an advantage of low cost over the existing object detection methods that explicitly require preparing ground-truth bounding boxes, which might be very expensive. Since the number of ground-truth bounding boxes is usually limited due to its high cost, the object detection methods tend to show bad performance in terms of the class accuracy and cannot detect any lesions in some cases. We have shown that our model outperforms object detection methods such as SSD and Faster R-CNN using 1,057 bounding boxes annotated by ophthalmologists for the dry AMD and wet AMD cases in terms of predicting the class labels. For future work, we will investigate the optimization of hyperparameters {*β*_*i*_} for better performance and the real-time EG algorithm for supporting real-time CAD systems.

## Supporting information

S1 Fig Qualitative analysis S1: Dry AMD case. (a) is showing the input image that we feed the CNN model, (b) is showing overlay visualized attribution map and bounding boxed localization image from the guided-backpropagation method, (c) is showing ground truth image defined by skilled ophthalmologist, (d) is showing overlay visualized attribution map and bounding boxed localization image from the IG algorithm, and (e) is indicating the result from the our method (EG). Our model predicts the input image as dry AMD with score of 0.982668. As seen in this figure, EG generates bounded boxes with a more ordered along with retinal layer than guided-backpropagation or IG.
(TIF)

S2 Fig Qualitative analysis S2: Wet AMD (with anti-VEGF injection required) case. (a) is showing the input image that we feed the CNN model, (b) is showing overlay visualized attribution map and bounding boxed localization image from the guided-backpropagation method, (c) is showing ground-truth image defined by skilled ophthalmologist, (d) is showing overlay visualized attribution map and bounding boxed localization image from the IG algorithm, and (e) is indicating the result from the our method (EG). Our model predicts the input image as wet AMD (with anti-VEGF injection required) with score of 0.99987. As seen in this figure, EG produces the more clearer overlay map and focuses on fluids in the image where guided-backpropagation method and IG algorithm do not.
(TIF)

S3 Fig Qualitative analysis S3: Wet AMD (with anti-VEGF injection required) case. (a) is showing the input image that we feed the CNN model, (b) is showing overlay visualized attribution map and bounding boxed localization image from the guided-backpropagation method, (c) is showing ground truth image defined by skilled ophthalmologist, (d) is showing overlay visualized attribution map and bounding boxed localization image from the IG algorithm, and (e) is indicating the result from the our method (EG). Our model predicts the input image as wet AMD (with anti-VEGF injection required) with score of 0.99941. As seen in this figure, EG produces the more specific overlay map and detect a fluid in the image where guided-backpropagation method and IG algorithm cannot.
(TIF)

S4 Fig Examples of ground-truth bounding boxes containing background pixels. (TIF)
