# Supplementary figures and images for: Weakly supervised lesion localization for age-related macular degeneration detection using optical coherence tomography images
Source: PLoS One. 2019 Apr 5;14(4):e0215076. doi: 10.1371/journal.pone.0215076 (PMC6450633; doi:10.1371/journal.pone.0215076)

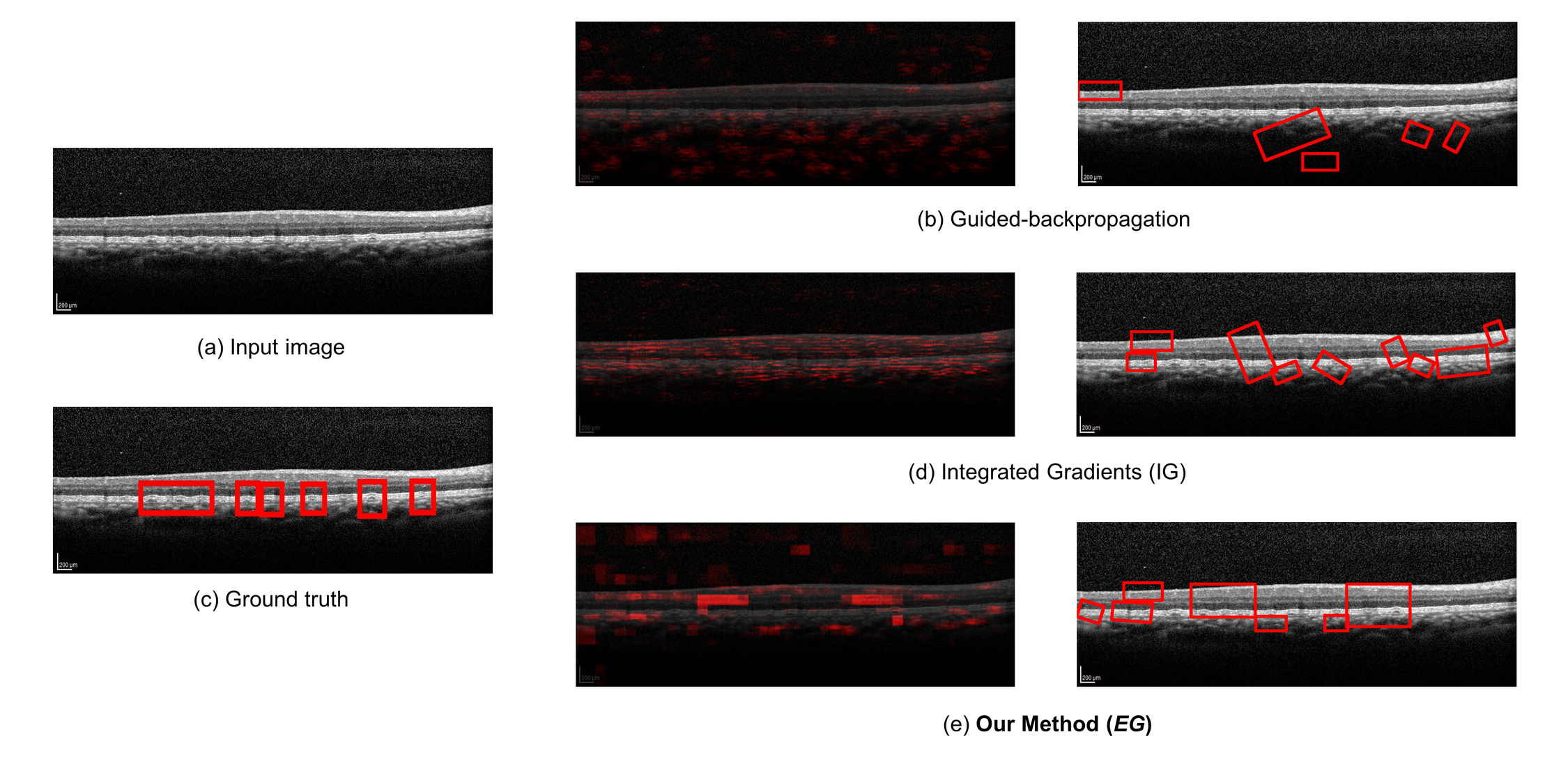

Supplement: S1 Fig — (a) is showing the input image that we feed the CNN model, (b) is showing overlay visualized attribution map and bounding boxed localization image from the guided-backpropagation method, (c) is showing ground truth image defined by skilled ophthalmologist, (d) is showing overlay visualized attribution map and bounding boxed localization image from the IG algorithm, and (e) is indicating the result from the our method (EG). Our model predicts the input image as dry AMD with score of 0.982668. As seen in this figure, EG generates bounded boxes with a more ordered along with retinal layer than guided-backpropagation or IG. (TIF) [file pone.0215076.s001.tif]

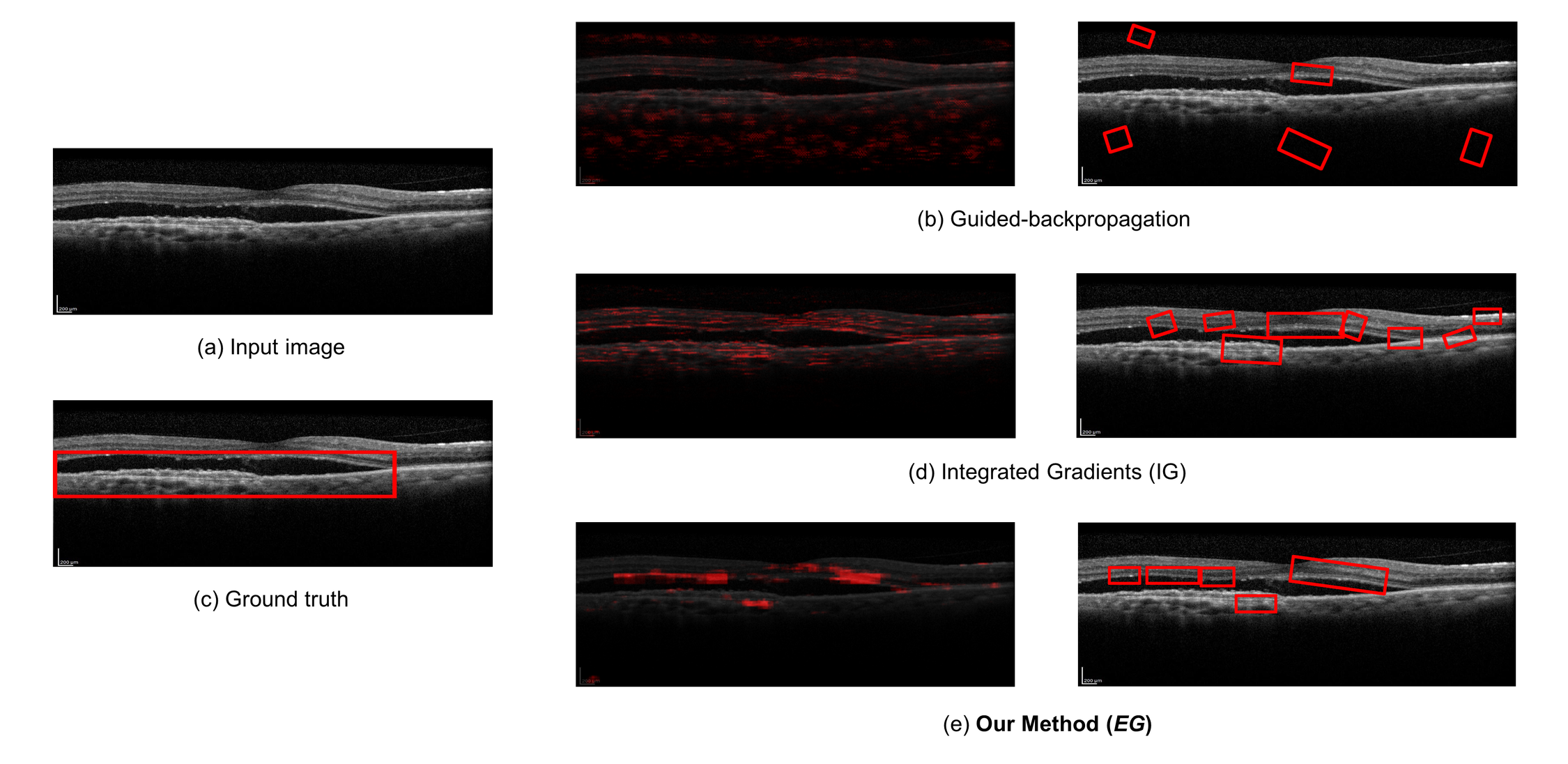

Supplement: S2 Fig — (a) is showing the input image that we feed the CNN model, (b) is showing overlay visualized attribution map and bounding boxed localization image from the guided-backpropagation method, (c) is showing ground-truth image defined by skilled ophthalmologist, (d) is showing overlay visualized attribution map and bounding boxed localization image from the IG algorithm, and (e) is indicating the result from the our method (EG). Our model predicts the input image as wet AMD (with anti-VEGF injection required) with score of 0.99987. As seen in this figure, EG produces the more clearer overlay map and focuses on fluids in the image where guided-backpropagation method and IG algorithm do not. (TIF) [file pone.0215076.s002.tif]

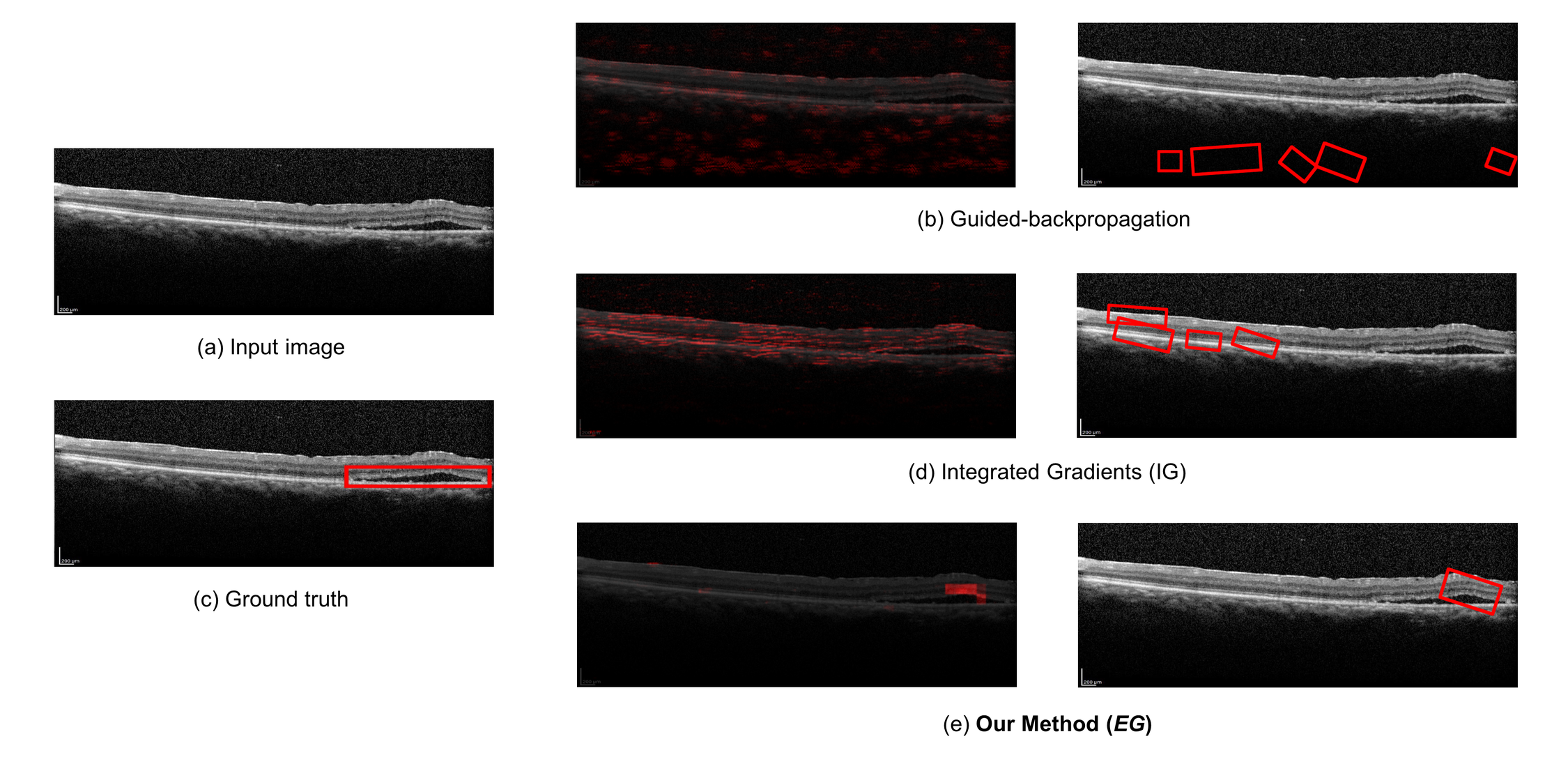

Supplement: S3 Fig — (a) is showing the input image that we feed the CNN model, (b) is showing overlay visualized attribution map and bounding boxed localization image from the guided-backpropagation method, (c) is showing ground truth image defined by skilled ophthalmologist, (d) is showing overlay visualized attribution map and bounding boxed localization image from the IG algorithm, and (e) is indicating the result from the our method (EG). Our model predicts the input image as wet AMD (with anti-VEGF injection required) with score of 0.99941. As seen in this figure, EG produces the more specific overlay map and detect a fluid in the image where guided-backpropagation method and IG algorithm cannot. (TIF) [file pone.0215076.s003.tif]

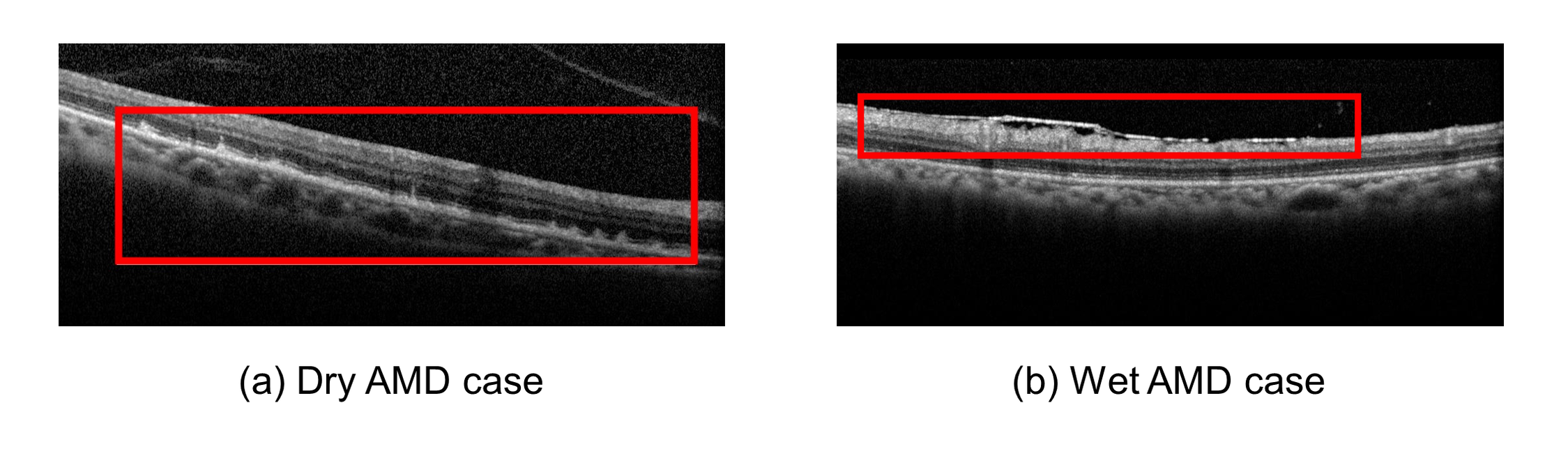

Supplement: S4 Fig — (TIF) [file pone.0215076.s004.tif]
